# The Cytotoxicity of Benzaldehyde Nitrogen Mustard-2-Pyridine Carboxylic Acid Hydrazone Being Involved in Topoisomerase II**α** Inhibition

**DOI:** 10.1155/2014/527042

**Published:** 2014-06-05

**Authors:** Yun Fu, Sufeng Zhou, Youxun Liu, Yingli Yang, Xingzhi Sun, Changzheng Li

**Affiliations:** ^1^Department of Molecular Biology & Biochemistry, Xinxiang Medical University, 601 Jinsui Road, Xinxiang, Henan 453003, China; ^2^Clinical Skill Training Center, Xinxiang Medical University, Xinxiang, Henan 453003, China

## Abstract

The antitumor property of iron chelators and aromatic nitrogen mustard derivatives has been well documented. Combination of the two pharmacophores in one molecule in drug designation is worth to be explored. We reported previously the syntheses and preliminary cytotoxicity evaluation of benzaldehyde nitrogen mustard pyridine carboxyl acid hydrazones (BNMPH) as extended study, more tumor cell lines (IC_50_ for HepG2: 26.1 ± 3.5 **μ**M , HCT-116: 57.5 ± 5.3 **μ**M, K562: 48.2 ± 4.0 **μ**M, and PC-12: 19.4 ± 2.2 **μ**M) were used to investigate its cytotoxicity and potential mechanism. *In vitro* experimental data showed that the BNMPH chelating Fe^2+^ caused a large number of ROS formations which led to DNA cleavage, and this was further supported by comet assay, implying that ROS might be involved in the cytotoxicity of BNMPH. The ROS induced changes of apoptosis related genes, but the TFR1 and NDRG1 metastatic genes were not obviously regulated, prompting that BNMPH might not be able to deprive Fe^2+^ of ribonucleotide reductase. The BNMPH induced S phase arrest was different from that of iron chelators (G1) and alkylating agents (G2). BNMPH also exhibited its inhibition of human topoisomerase II**α**. Those revealed that the cytotoxic mechanism of the BNMPH could stem from both the topoisomerase II inhibition, ROS generation and DNA alkylation.

## 1. Introduction

Cancer is an important health issue that caused million deaths. Localized cancer can be resected by surgery, but metastasis cancer has to be treated systematically by chemotherapy in combination with radiotherapy [1]. However, chemotherapeutic agents used are cytotoxic in genetics and have some side effects due to lack of selectivity toward tumor cells. One approach to improving the therapeutic effectiveness and decreasing systemic side effects is through the design of anticancer drug based on the tumor cell characteristic. It is well known that iron is an essential element and that it plays a crucial role in cellular proliferation and DNA synthesis. In general neoplastic cells have high requirement for iron for their significantly elevated expression of the transferrin receptor 1 (TfR1) [2]. Similarly, the higher levels of the Fe-containing enzyme, ribonucleotide reductase (RR), for DNA synthesis are also found in neoplastic cells. In addition, it has been shown that cancer cells also take up more copper (Cu) than normal cells and use this metal for angiogenesis and metastasis [3]. Based on the crucial roles of these metals, developing novel Fe and Cu chelators has become a promising anticancer strategy [4]. Desferrioxamine (DFO) and Dp44mT are examples of this kind of metal chelators and have excellent antitumor activity [5, 6].

Nitrogen mustard as alkylating agent is one of the most useful drugs in cancer chemotherapy. Due to the lack of selectivity and toxic side effect as well as resulting drug resistance, carcinogenesis and mutation, its applications are limited. To improve its antitumor activity, many strategies are tried to design specific target alkylating agents, such as DNA-directed alkylating agents which were made by linking DNA-affinic molecules (carriers) to a nitrogen mustard pharmacophore (warhead) for these DNA-affinic molecules also exhibit topoisomerases I and II inhibition [7, 8]. In those strategies the aromatic nitrogen mustards are mostly used as alkylating pharmacophore (such as benzoic acid nitrogen mustard) as they are effective against quiescent cell and trend to be less susceptible to induced resistance than most anticancer drugs.

Combining two pharmacophores into one molecule could make it with dural targets, which may both enhance its cytotoxicity and reduce resistance when administrated. As mentioned above, iron chelator can interfere with iron metabolism by depriving iron either from enzymes (proteins) or from labile iron pool. And tumor cells require more iron for their growth. The drug tethering both alkylating and chelating groups is another option to attempt improving its biological activity. We made some hydrazones by condensation of pyridine carboxyl acid hydrazides with benzaldehyde nitrogen mustard [9, 10], indicating that the hydrazones indeed enhance their antitumor activities compared to the parent compounds. As extended study, more cancer cell lines were used to explore the potential mechanism; the results indicated that the hydrazone exerts its cytotoxicity via disturbing host cell cycle, inhibiting topoisomerase II and regulating apoptosis related genes. However, the introduction of chelating group which desires to regulate metastasis genes seems not to disturb the iron metabolism.

## 2. Materials and Methods

All reactants and solvents were AR grade. MTT, ethidium bromide (EB), RPMI-1640, and agarose were purchased from Sigma.

### 2.1. Preparation of Benzaldehyde Nitrogen Mustard-2-Pyridine Carboxyl Acid Hydrazone (BNMPH)

**Figure 1:**
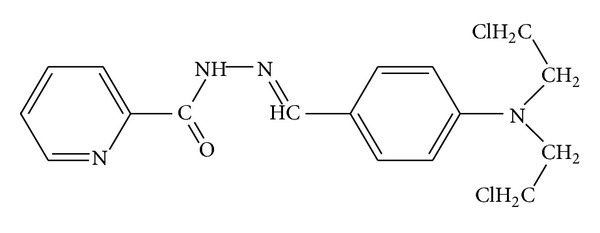
The chemical structure of BNMPH.

Benzaldehyde nitrogen mustard-2-pyridine carboxyl acid hydrazone was made as described [9]. The BNMPH structure is shown in Figure 1.

### 2.2. The Cytotoxicity of the BNMPH

The stock solution of BNMPH was prepared in DMSO; 1 mg/mL cisplatin (in physiological saline) was used as positive control. The BNMPH solution was diluted to the required concentration with culture when used. The Human colorectal carcinoma cell line (HCT-116), human erythromyeloblastoid leukemia cell line (K562), and liver carcinoma cells (HepG2) were cultured in RPMI 1640 medium supplemented with 10% fetal calf serum (mycoplasma free FCS) and antibiotics. The rat pheochromocytoma cell line (PC-12) was cultured as HepG2 except 10% horse serum supplemented. The cells collected from exponential phase (2 × 10^4^/mL) were seeded equivalently into 96-well plate and the various amounts of BNMPH were added after the cells adhered. Following 48 h incubation at 37°C in a humidified atmosphere of 5% CO_2_, 10 *μ*L MTT solution (1 mg/mL) was added to each well, followed by further incubation of 4 h. The cell culture was removed by aspiration, and 100 *μ*L DMSO was added in each well to dissolve the formazan crystals. The measurement of absorbance of the solution that was related to the number of live cells was performed on a microplate reader (MK3, Thermo Scientific) at 492 nm. Percent growth inhibition was defined as percent absorbance inhibition within appropriate absorbance in each cell line. The same assay was performed in triplet.

### 2.3. ROS Detection and DNA Cleavage Caused by BNMPH

H_2_DCF-AM was converted to dichlorofluorescein (DCF) according to reports by Jakubowski and Bartosz [11]. Briefly, 0.25 mL of 2 mM H_2_DCF-AM in absolute ethanol was added to 2.0 mL of 10 mM NaOH and allowed to stand at room temperature for 30 min. The hydrolysate was then neutralized with 10 mL of 25 mM sodium phosphate buffer (pH 7.2) and kept on ice for use. Reaction buffer (50 mM sodium phosphate buffer, pH 7.4) containing 0.4 *μ*M DCF was mixed with 25 *μ*L of 1 mM (NH_4_)_2_Fe(SO_4_)_2_ or 1 mM BNMPH or 0.2 mL of 4 mM H_2_O_2_ in a total volume of 4.0 mL. The fluorescence of each sample was measured in FC-960 spectrofluorimeter (excitation at 488 and emission at 520 nm). The measurements were conducted at room temperature.

The ROS induced DNA cleavage was assessed as described [12], and the concentration was used in the assay as indicated in Figure 3(b).

### 2.4. Comet Assay

The single-cell gel electrophoresis (comet assay) was adapted from the method as described [13]. HepG2 cells treated with or without 50 *μ*M of BNMPH after 48 h incubation in a humidified atmosphere of 5% CO_2_ were harvested by centrifugation at 1000 rpm and then embedded in 0.5% low-melting-point agarose at a final concentration of 10^4^ cells/mL. 20 *μ*L of this cellular suspension was then spread onto duplicate frosted slides that had previously been covered with 1% normal melting point agarose as a basal layer. Slides were allowed to solidify for 10 min at 4°C before being placed in lysis buffer for 1 h (2.5 M NaCl, 0.1 M ethylene diamine tetraacetic acid (EDTA), 0.01 M Tris, 1% Triton X-100, and 10% dimethyl sulfoxide (DMSO), pH 10). After lysis, the slides were transferred into alkaline buffer for 40 min (0.001 M EDTA, 0.3 M NaOH, pH > 13) to allow the DNA to unwind before migration at 0.66 V/cm and 300 mA for 30 min. All these steps were performed in the dark. After neutralisation in 0.4 M Tris HCl pH 7.4, slides were stored at 4°C until analysis within the following 24 h. Before analysis, the slides were stained with ethidium bromide (EB) (20 *μ*g/mL) and covered with a cover slip. The photograph was taken on fluorescent microscopy.

### 2.5. Alkaline Agarose Gel Shift Assay

DNA cross-linking of BNMPH was assayed by alkaline agarose gel electrophoresis as described [8]. In brief, purified pUC18 plasmid DNA (1 *μ*g) was mixed with various concentrations (12.5–50 *μ*M) of BNMPH in 10 *μ*L reaction buffer (10 mM sodium phosphate, pH 7.4 containing 3 mM NaCl and 1 mM EDTA). The reaction mixture was kept at 37°C for 2 h. Finally, the plasmid DNA was digested to linear DNA with Xba I and followed by phenol extraction and ethanol precipitation. The DNA pellets were dissolved in alkaline buffer (0.5 M NaOH, 10 mM EDTA), mixed with alkaline loading dye, and then electrophoresed on a 1% alkaline agarose gel with NaOH-EDTA buffer at 4°C. The electrophoresis was carried out at 30 V for 2 h. After staining the gels with an ethidium bromide solution, the DNA was then visualized on Toucan 360 gel imager (version 3.2.1 software).

### 2.6. Thermal Denaturation

The DNA melting experiments were carried out in the temperature range from 25 to 95°C with a Shimadzu circulation bath, monitoring the absorbance of Ct-DNA at 260 nm in the absence or presence of BNMPH. The melting temperature (Tm) of DNA is defined as the temperature at which 50% of double strand becomes single stranded. It was determined as the midpoint of the optically detected transition curves [14].

### 2.7. Cell Cycle Analysis

HepG2 cells (1 × 10^5^) were seeded in a six-well plate. After 24 h of incubation at 37°C (5% CO_2_), the medium was changed with fresh, supplemented or not (control) with BNMPH (6, 12 *μ*M). After 24 h of incubation, cells were harvested with trypsin, washed by PBS, fixed in 70% ethanol, and stored at −20°C. The cellular nuclear DNA was stained by propidium iodide (PI). Briefly, after removing the 70% ethanol, the cells were washed with PBS and then suspended in 0.5 mL PBS containing 50 *μ*g/mL PI and 100 *μ*g/mL RNase. The cell suspension was incubated at 37°C for 30 min. DNA flow cytometry was performed in duplicate with a FACScalibur flow cytometer (Becton Dickinson, USA). Each sample's 10000 events were collected and fluorescent signal intensity was recorded and analyzed by CellQuest and Modifit (Becton Dickinson, USA).

### 2.8. The Effect of the BNMPH on Apoptotic and Metastatic Gene Regulation by RT-PCR

To explore the underlying mechanism, the HepG2 cells were treated with the BNMPH. The RT-PCR was conducted to determine both the changes of apoptotic genes such as p53, caspase-3, and caspase-8 and metastatic genes, such as TFR1 and NDRG1 at mRNA level. The gene expressions investigated were measured after 24 h incubation with different concentrations of BNMPH. Total RNA was extracted from the cells using Trizol reagent (Sangon, Shanghai, China) according to the manufacturer's protocol. Three micrograms of total RNA was used for reverse transcription in a total volume of 20 *μ*L with the M-MLV reverse transcriptase system (LifeFeng Biotechnology Co., Shanghai, China). 2 *μ*L cDNA was subsequently amplified in a total volume of 20 *μ*L using the 2xTaq PCR kit (LifeFeng Biotechnology Co., Shanghai, China) following conditions recommended by the manufacturer. The sense and antisense primers (primers were synthesized by Shanghai General Bioengineering Co. in the study, Shanghai, China) for beta actin were 5′-ACACTGTGCCCATCTACGAGG-3′ and 5′-CGGACTCGTCATAC TCCTGCT-3′ (615 bp). They were used as an internal control; the sense and antisense primers for caspase-3 were 5′-GAAGCGAATCAAT GGACTCTGG-3′ and 5′-ACA TCACGCAT CAATTCCAC AA-3′ (241 bp); the sense and antisense primers for caspase-8 were 5′-AAGT TCCTGAGCCTGGACTACAT-3′ and 5′-ATTTGAGCCC TGCCTGGTGTCT-3′ (227 bp); the sense and antisense primers for p53 were 5′-GTCTACCTCCCGCC ATAA-3′ and 5′-CATCTCCCAAACATCCCT-3′ (316 bp); the sense and antisense primers for NDRG1 were 5′-CCCTCGCGTTA GGCAGGTG A-3′ and 5′-AGGGGTA CA TGTACC CTGCG-3′ (370 bp); the sense and antisense primers for TFR1 were 5′-TCAGGTCAAAGACAG CGCTCAAAACTC-3′ and 5′-AG TCTCCTTCCATATTCCCAAACAGCTTTT-3′ (358 bp), respectively. The RT-PCR was performed on Nexus Gradient Mastercycler (Eppendorf). The cycling conditions were 94°C for 5 min, followed by 30 cycles of 94°C for 30 s, 53–56°C for 30 s,72°C for 1 min, and a final extension of 72°C for 10 min. PCR products were separated on the 1.5% agarose gel viewed by ethidium bromide staining. These data were acquired with Toucan 360 gel imager (version 3.2.1 software).

### 2.9. DNA Relaxation of Supercoiled pUC18 DNA

This assay was performed following the procedure reported [15, 16]. The reaction mixture (20 *μ*L) contained relaxation buffer (50 mM Tris-HCl (pH 8.0), 0.5 mM EDTA, 1 mM DTT, 50 mM NaCl, 10 mM MgCl_2_, and 30 *μ*g/mL BSA, 5% glycerol), 0.3 *μ*g of supercoiled pUC18 plasmid DNA, and increasing concentrations of the BNMPH. The reaction was initiated by adding 1 unit of human topoisomerase II*α* (Sigma) and incubated at 37°C for 45 min. The reaction was terminated by adding 5 *μ*L of stopping buffer (10% SDS, 25 mM EDTA, 0.025% bromophenol blue, and 40% glycerol). The reaction products were analyzed by electrophoresis on 1% agarose gel using a TBE buffer (89 mM Tris-HCl, 89 mM boric acid, and 62 mM EDTA) at 30 V for 3 h, stained by ethidium bromide (0.5 *μ*g/mL), and photographed by a Toucan 360 gel imager (version 3.2.1 software).

### 2.10. Molecular Docking Studies

The structure of human type II DNA topoisomerase with DNA and etoposide (3QX3) was obtained from RCSB Protein Data Bank [17]. The BNMPH was generated from Chemdraw (Chemdraw Ultra 8.0) and the energy minimization was conducted by Chem3D (Ultra 8.0) [18]. The resulting models were displayed in PyMOl (The PyMOL Molecular Graphics System, Version 1.4.1, Schrödinger, LLC).

Molecular docking studies were performed by AutoDock Vina and AutoDock Tools based on the recommended procedure [19]. Grid boxes were set to the center of etoposide model, and the grid box size for BNMPH was set to 22, 24, and 28 for *X*, *Y*, and *Z* axes, respectively. The BNMPH was set as a flexible ligand by using the default parameters of the AutoDock Tools. The optimal conformation of the ligand was generated by Autodock Vina.

## 3. Results 

### 3.1. The Cytotoxicity of the BNMPH

**Figure 2:**
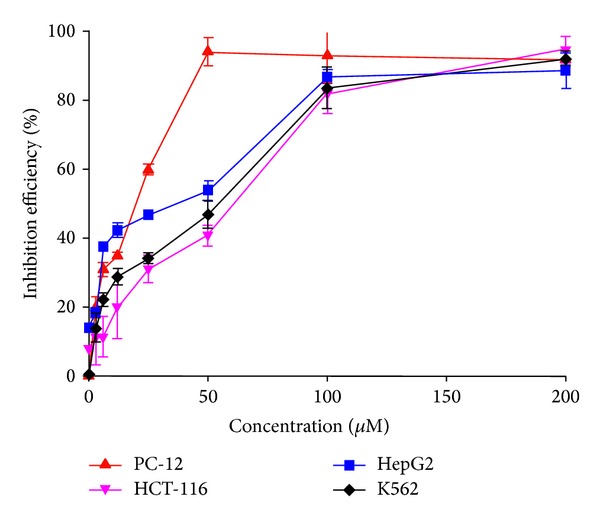
Cytotoxicities of BNMPH in the indicated cell lines (IC_50_: 57.5 ± 5.0 *μ*M for HCT-116; 19.4 ± 2.2 *μ*M for PC-12; 26.1 ± 3.5 *μ*M for HepG2; and 48.2 ± 4.0 *μ*M for K562).

The previous study showed that the BNMPH can chelate many transition metals such as iron, copper, and zinc ion to form metal complexes; however, the evaluation of it and its metal complexes in anticancer activity was not fully conducted. To get insight of more information, BNMPH was evaluated against different tumor cell lines (HepG2, HCT-116, PC-12, and K562) by MTT method. The dose-response curves of BNMPH determined against the investigated cells are depicted in Figure 2. As shown in Figure 2, BNMPH could inhibit all the cell lines and had moderate growth inhibition in the K562 (48.2 ± 4.0 *μ*M) and HCT-116 (57.5 ± 5.3 *μ*M), stronger inhibition in HepG2 hepatocellular carcinoma cell line (26.1 ± 3.5 *μ*M, accordingly the IC_50_ of cisplatin obtained by our lab was 18.9 ± 2.4 *μ*M [20]), and significant inhibition for PC-12 cell line (19.4 ± 2.2 *μ*M).

### 3.2. BNMPH Induced ROS Generation

**Figure 3:**
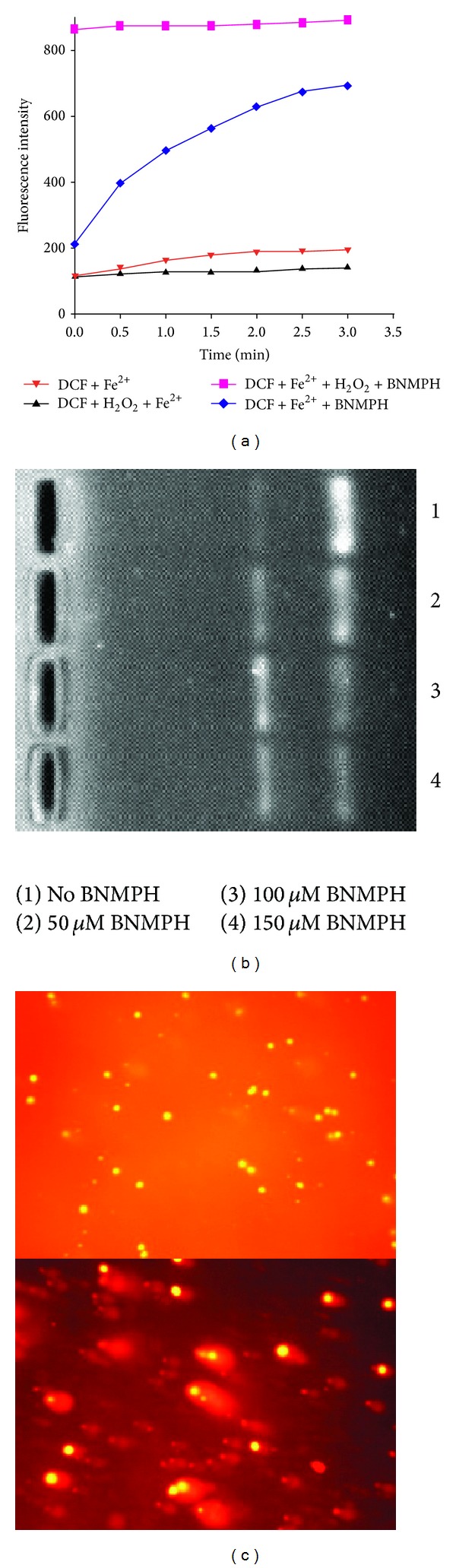
BNMPH redox activity and induction of DNA fragmentation. (a) ROS generation by Fenton reaction; the ROS product was measured by DCF fluorescence. (b) The ROS induced DNA fragmentation in the presence or absence of BNMPH; the fragmented DNA was separated by agarose gel electrophoresis and visualized by EB staining, and the concentration is as indicated. (c) DNA fragmentation* in vivo* was evaluated by comet assay, top: control, and bottom: in the presence of 50 *μ*M BNMPH.

Many chemotherapeutic agents exhibit their antitumor activity via formation of reactive oxygen species (ROS). To assess whether BNMPH involved in ROS formation, the ROS assay was performed. As shown in Figure 3(a), BNMPH promoted ROS formation compared to Fenton reaction with significant increasing (~6 times) base on the fluorescent intensity (Figure 3(a)). It was interesting that BNMPH could also induce oxygen free radical formation in a time dependent manner, which implied that the BNMPH could chelate ferrous ion, and the formed complex was redox activity. In view of highly reactive feature of ROS and ROS causing oxidative damage of DNA, the cleavage of pUC18 by BNMPH-Fe^2+^ complex was assessed by agarose gel electrophoresis. As shown in Figure 3(b), the supercoiled pUC18 was decreased in the presence of BNMPH, and accordingly the cleaved DNA was increased. So we speculated the cytotoxicity of BNMPH was correlated with ROS generation.

### 3.3. Cellular DNA Fragmentation* In Vitro*

ROS assay indicated that the BNMPH (or its iron complex) is redox active, and accordingly* in vivo* the ROS could cause genetic DNA breakage of host cell. To evaluate the potential effect of BNMPH on DNA integrity, the comet assay was conducted. As shown in Figure 3(c), BNMPH caused the cellular DNA breakage; the cometic tail of DNA is presented in the BNMPH treated K562 cells.

### 3.4. DNA Cross-Linking of BNMPH

**Figure 4:**
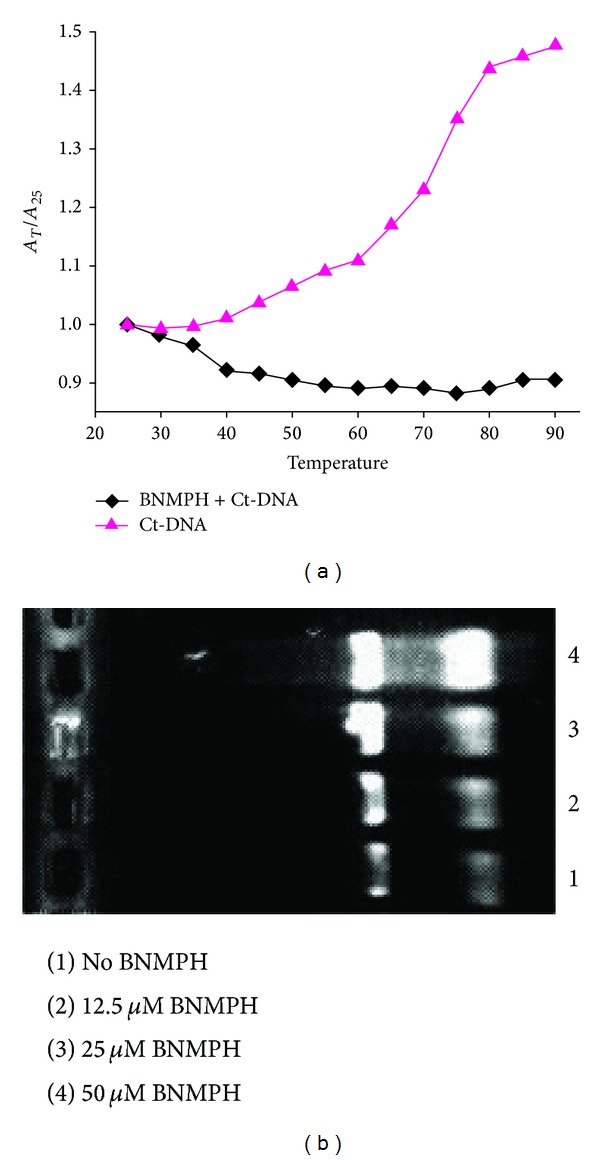
DNA alkylation of BNMPH. (a) BNMPH effect on DNA thermal denaturation. The flatted curve (black) indicated that the dissociation of double stranded DNA was blocked during temperature rising due to cross-linking. (b) BNMPH induced DNA cross-linking. The cross-linked DNA was separated by electrophoresis and visualized by EB staining, and the concentrations used are as indicated.

To assess DNA cross-linking capacity of BNMPH, pUC18 DNA was used to react with varied BNMPH concentration, and the reaction products were subjected to alkaline agarose gel electrophoresis after BamH1 digestion (Figure 4(b)) [21]. As shown in Figure 4(b), BNMPH was able to induce DNA interstrand cross-linking, suggesting that BNMPH induced DNA cross-linking may also contribute to its cytotoxicity.

### 3.5. Thermal Denaturation

To further confirm the action mode of BNMPH with DNA, the effect of it on melting point of Ct-DNA was conducted. As shown in Figure 4(a), with increasing the temperature the absorbance at 260 nm of Ct-DNA solution was increased with double-helix dissociation to single strands since heat damages those hydrogen bonds. The melting temperature (Tm) of Ct-DNA was 72°C in the absence of BNMPH; however, the shift of the curve of Ct-DNA in the presence of BNMPH did not occur; conversely, it was fully flatted, indicating that the dissociation of double-helical Ct-DNA was blocked. That was also indicative of covalent bond formed between stranded Ct-DNA.

### 3.6. BNMPH Gene Regulation

**Figure 5:**
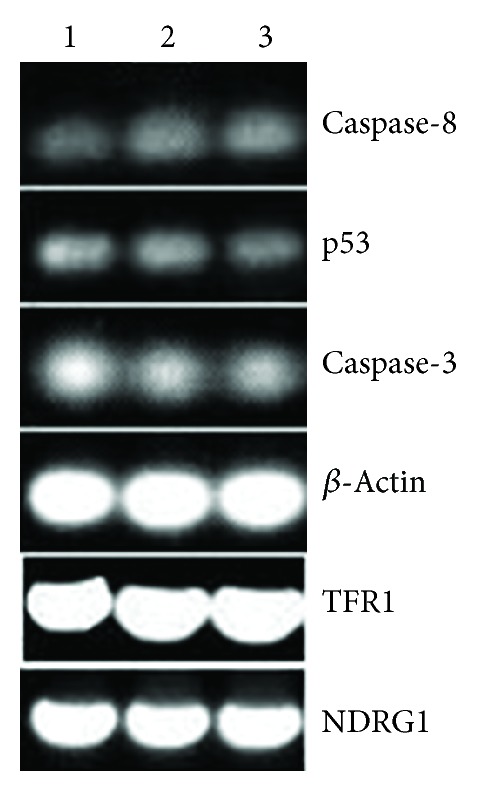
The effect of BNMPH on gene regulation. 1: 12 *μ*M BNMPH; 2: 6 *μ*M BNMPH; 3: control, the genes in the figure as indicated.

ROS play a crucial role in cell growth and apoptosis. BNMPH induced production of ROS* in vitro* encouraged us to investigate its gene regulation. So, the RT-PCR was conducted to determine the changes of apoptotic genes, such as p53, caspase-3, and caspase-8 after the HepG2 cells were treated with BNMPH. As shown in Figure 5, the response of caspase-3 and caspase-8 was not parallel (Figure 5) and that of caspase-3 and p53 was increased significantly, but caspase-8 was not upregulated. In general, N-myc downstream-regulated gene 1 (NDRG1), a metastasis suppressor, is upregulated by cellular iron depletion [22]. In view of the potent iron chelating ability of BNMPH, the effect of it on regulation of metastatic and iron related gene, NDRG1 and TFR1 were also assessed by RT-PCR. Beyond our expectation, those genes were not affected by exposure of the BNMPH at the investigated concentrations. It might be indicative that the BNMPH was not involved in iron deprivation from ribonucleotide reductase as those inhibitors usually cause a G1/S or G2 arrest.

### 3.7. BNMPH Induced Cell Cycle Arrest at S Phase

**Figure 6:**
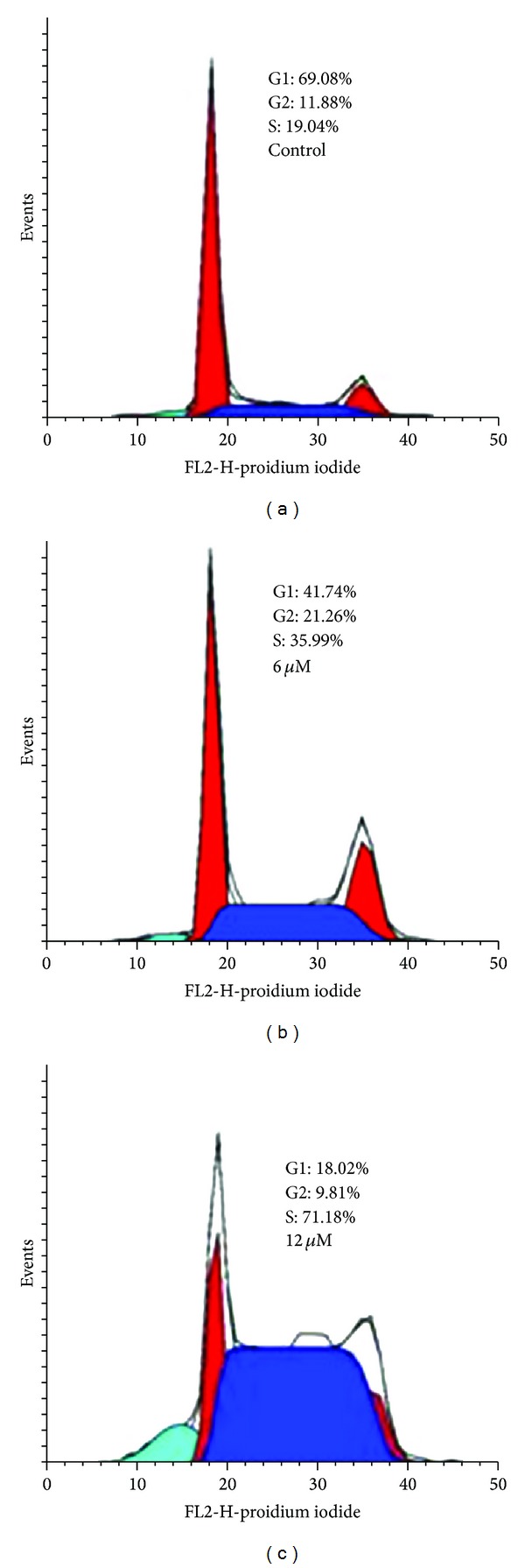
BNMPH induced cell cycle arrest. (a) Control; (b) 6 *μ*M BNMPH; and (c) 12 *μ*M BNMPH, the cell distribution in the figure as indicated.

It had been reported that DNA alkylating agents induced cell cycle delay and arrested the cell cycle progression predominantly at the G2/M boundary [23]. We therefore evaluated the effect of BNMPH on the cell cycle distribution using propidium iodide staining and flow cytometry. As shown in Figure 6, BNMPH caused an accumulation of cells in the S phase of the cell cycle. The percentage of cells at the S phase significantly increased from 19.04 to 35.99 and to 71.18% after treatment with 6 and 12 *μ*M BNMPH, respectively. The ability of BNMPH to induce S cell cycle arrest in wildtype p53 containing HepG2 cells is also indicative of its p53-dependent mechanism [24], which was consistent with the upregulation of p53 in RT-PCR experiment. It was surprising that there was a difference in cell cycle arrest between BNMPH and the other nitrogen mustard containing derivatives [21, 23], which might reflect the sensitivity of the cells used to the test compound or dose dependent or different mechanism.

### 3.8. DNA Relaxation Inhibition

**Figure 7:**
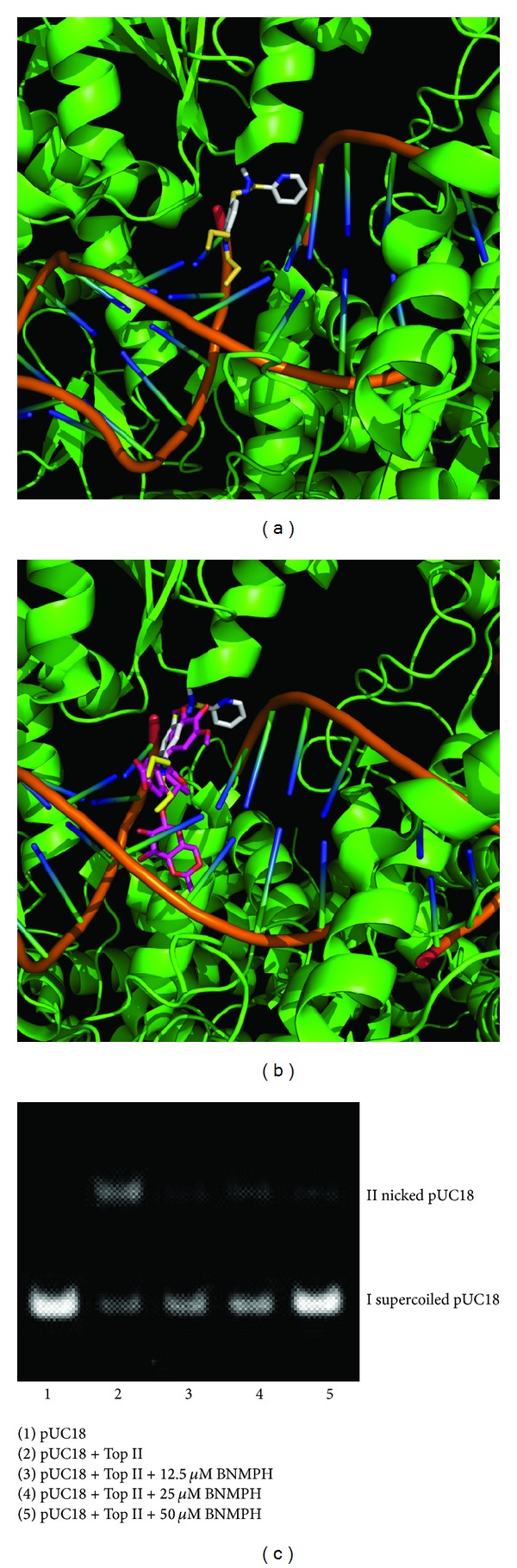
Topoisomerase II inhibition of BNMPH. (a) The docked BNMPH in human topoisomerase II-DNA complex. (b) Comparison of BNMPH and etoposide in topoisomerase II complex. (c) Human topoisomerase II*α* inhibition of BNMPH. The concentration is used as indicated. At 50 *μ*M BNMPH almost blocked the topoisomerase catalyzed DNA relaxation.

**Figure 8:**
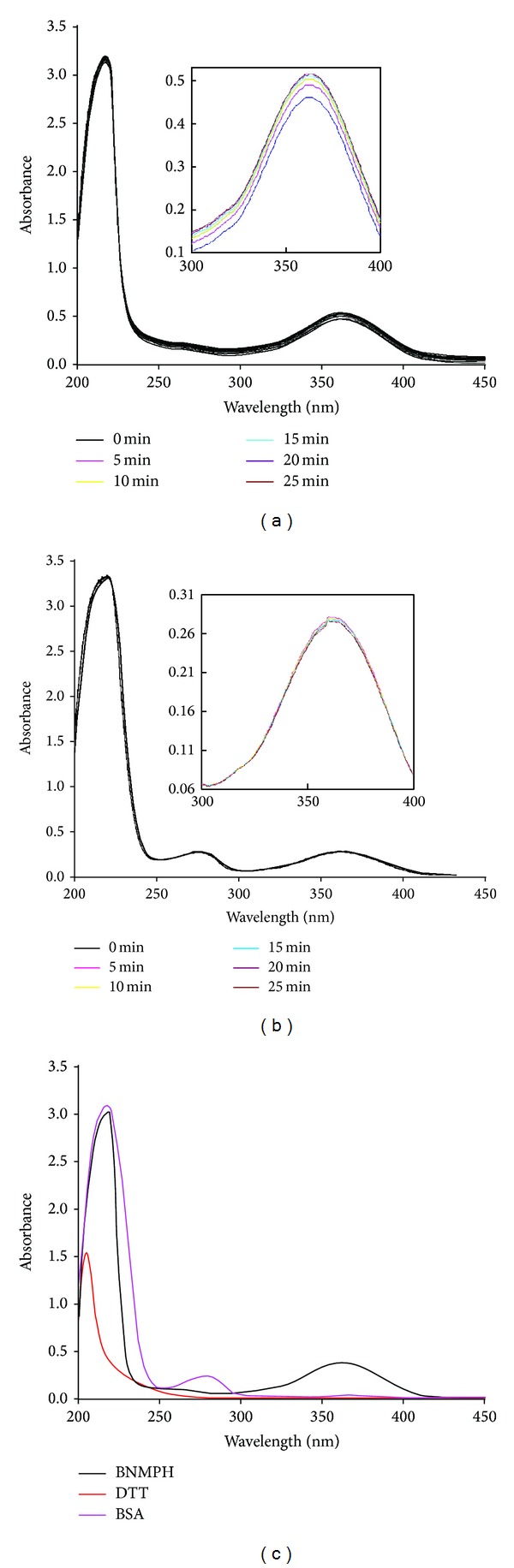
BNMPH reactivity with DTT and BSA. (a) The absorbance spectral changes of DTT after addition of 26 *μ*M BNMPH to 500 *μ*M DTT (pH 8.0, 37°C). The spectrum was collected after the mixing, and subsequent spectrum at 5 min intervals was recorded. The insert showed a series of increases during the 25 min period, indicating that BNMPH can alkylate thiol group. (b) Spectrum of BNMPH (13 *μ*M) reacted with thiol of BSA (5 *μ*M) in pH 8.0 buffer at 37°C; the insert showed no obvious changes during the 25 min period, indicating that BNMPH may react with thiol group of BSA at very low rate.

The effects of the BNMPH on topoisomerase II-mediated double strand DNA relaxation was carried out to test whether the BNMPH was topoisomerase II poisons. As shown in Figure 7(c), BNMPH was effective in preventing the conversion of supercoiled pUC18 to nicked closed circular DNA in a concentration dependent manner, indicating that it may inhibit topoisomerase II catalytic (relaxation) activity (Figure 7(c)). It almost eliminated the topoisomerase activity at 50 *μ*M. On the other hand, it has been well documented that nitrogen mustard reacts preferably with sulfhydryl compounds, so the BNMPH reactivity with both DTT and BSA was investigated by spectral method, as shown in Figures 8(a) and 8(b). BNMPH indeed reacted with DTT, but the rate was not fast as the literature reported. We also observed that the rate of BNMPH which reacted with BSA was quite slow. In view of excess DTT (0.5 mM) used in the topoisomerase inhibition assay, some of BNMPH could react with DTT; however, it was a little surprising that BNMPH still showed the capacity in topoisomerase inhibition, so we speculated BNMPH exerted the inhibition might not be due to its reactivity with cysteine of topoisomerase, instead of interaction with catalytic (relaxation) domain.

### 3.9. Molecular Docking Studies

The topoisomerase inhibition of BNMPH* in vitro* demonstrated that BNMPH might be poisonous to topoisomerase Ii*α*; however, many interaction modes could have occurred, that is, via alkylation of cysteine or binding at cleavage site or competition ATP binding site of topoisomerase complex. Due to slower reactivity of BNMPH with -SH group, especially with -SH group from protein, molecular docking was performed to assess possible interaction of BNMPH with topoisomerase. Based on the recommended procedure, the ligand free type II topoisomerase derived from 3QX3 (PDB ID: 3QX3) was chosen as receptor, and the BNMPH was used as ligand. The input pbdqt files were generated by Autodock tool, and the optimal conformations of ligand were calculated on Autodock Vina. The calculated data indicated that the docked BNMPH had a −8.9 kcal/mol affinity energy with the DNA-topoisomerase complex. The orientation of the BNMPH and comparison of it with etoposide are shown in Figure 7(a) and Figure 7(b), respectively.

## 4. Discussion

Anticancer agents, such as paclitaxel, cisplatin, and doxorubicin induce apoptosis in most of the cancer cells through excess ROS formation [25, 26]. Among those drugs, some have potential metal chelating ability, which usually are involved in ROS generation. As an attempt, we designed the BNMPH tethered both alkylating and chelation group in order to improve the drug's cytotoxicity. Previous study has shown that the BNMPH can chelate many transition metals, such as iron, copper, and zinc ion and exhibit definite biological activity. However, its anticancer activity, especially the underlying mechanism, was less concerned. Fenton reaction, a well-documented ROS generation system, can be used to determine whether BNMPH is involved in ROS production. Our data indicated that the BNMPH-iron complex is redox active; it can promote both production of hydroxyl and oxygen free radical* in vitro*. This encourages us to investigate the effect of BNMPH on cellular DNA integrity* in vivo*. It is well known that the ROS have ability to cause cellular DNA fragmentation, and comet assay is a sensitive method for quantifying and analyzing DNA damage and is widely used in evaluation of genotoxicity and effectiveness. So, comet assay may provide useful information to determine whether the cytotoxicity exhibited BNMPH was involved in ROS generation. As expected, BNMPH indeed induced cellular DNA cleavage at higher concentration, indicating BNMPH's cytotoxicity may partly stem from ROS biological effect. It also prompts that iron chelating ability of BNMPH makes contribution to ROS generation; in other words, redox active BNMPH-Fe complex might be one of the poisons to host cell.

Nitrogen mustard derivatives as DNA alkylating agents can alkylate DNA via interstrand or intrastrand cross-linking (ICL), leading DNA lesions. It has been accepted that as a mechanism of inhibition or arrest of synthesis of DNA, other interactions, such as between DNA and proteins, including DNA polymerase at replication fork site may be also involved. BNMPH tethering an aromatic mustard could have similar function. As expected, the DNA alkylating ability of BNMPH was confirmed both by alkaline agarose gel shift assay and also the thermodenaturation of CT-DNA, in which the cross-linked DNA and blockage of DNA dissociation can be seen. In general, an ICL can lead to a stalled replication fork in S phase [27] and likely activate cell-cycle checkpoint and then arrest at late S to G2 [28] to repair the damaged DNA [29, 30]. It is beyond our prediction that BNMPH induced HepG2 cell at S phase arrest in a concentration dependent manner, which is a distinct difference from what the other alkylating agents did, but this situation was recently observed when HepG2 cells exposed to nitrogen mustard [31]. This may also indicate that there are other interactions, such as between DNA and proteins, including DNA polymerase, at replication fork site that may be also involved. Masta et al. demonstrated that nitrogen mustard can inhibit transcription and translation in a cell free system [32]. We also observed Taq DNA polymerase inhibition by BNMPH in PCR reaction, showing that the PCR products were decreased with increasing of BNMPH (data not shown). Thus, we speculate that BNMPH might be able to target transcription enzymes via alkylating cysteine, and consequently their enzymatic activities are lost or inhibited. On the other hand, to some extent, the reduction in fidelity of replication of DNA might contribute to the cytotoxicity of alkylating agents [33]. So the S phase arrest caused by BNMPH might be involved in the mentioned above inhibitions. To evaluate whether the topoisomerase affects the cell cycle, topoisomerase II*α* inhibition of BNMPH was investigated; the data clearly indicate that BNMPH indeed interfere with the topoisomerase function, which implies that dysfunction of topoisomerase may also contribute to the cell cycle delay, but the mode of topoisomerase inhibition of BNMPH, via alkylating SH, or other interactions is unclear. In view of reactivity of BNMPH with free -SH (DTT) and -SH from protein (BSA) is not strong (fast) based on our data. We speculate alkylating of -SH of topoisomerase might not be main contributor. To assess the possibility of “poising” of BNMPH to DNA-topoisomerase intermediate, molecular docking was conducted using available crystal structural data, revealing that the docked BNMPH has a moderate binding affinity with topoisomerase (−8.9 kcal/mol) compared to that redocked etoposide (−14.6 kcal/mol), indicating that the poising effect of BNMPH on DNA-topoisomerase intermediate cannot be excluded.

The involvement of reactive oxygen species (ROS) in induction of apoptosis of various cancer cells has been demonstrated in the literature [34–36]. The cytotoxicity of BNMPH could partly be due to ROS production. Under oxidative stress, the apoptosis related genes, such as caspase-3, caspase-8, and p53 will respond to the stress. As expected, the upregulation of p53 and caspase-3 was observed after 24 h of exposure, indicating that the cytotoxicity of BNMPH was involved in apoptosis. However, the changes of TFR1 and NDRG1 genes were not obvious, which might indicate that the cytotoxicity of BNMPH was not correlated with the iron deprivation from ribonucleotide reductase. The cell cycle analysis seemed to be supportive of this. Generally iron chelators induce upregulation of TFR, NDRG1, and G2 arrest [2, 5, 6]. The S phase delay induced by BNMPH may reflect the difference in coordination environment between BNMPH (bidentate) and iron chelators (tridentate). So, the BNMPH acquiring the iron for ROS generation might be from labile iron pool. Therefore, the cytotoxicity of BNMPH stems mainly from DNA alkylation, ROS generation, and topoisomerase inhibition.

## 5. Conclusion

In this work we evaluated the cytotoxicity and potent mechanism of BNMPH; the IC_50_ differences in the investigated cell lines reflect the sensitivity of BNMPH to cell lines. Like other nitrogen mustard derivatives, the BNMPH has the DNA alkylating ability to cross-link DNA. Iron chelating capacity of BNMPH also contributed to the ROS generation, which consequently induced apoptosis and DNA fragmentation of host cell. No changes of the Tfr1 and NDRG1gene expression and cell cycle arrest at S phase might indicate that the iron for ROS generation was not from ribonucleic reductase but from labile iron pool. Topoisomerase II*α* inhibition of BNMPH and molecular docking reveal the potent mechanism. Therefore, the conclusion can be drawn that the cytotoxicity of BNMPH may stem from ROS generation, DNA alkylating, and topoisomerase inhibition.
